# Nur1 Dephosphorylation Confers Positive Feedback to Mitotic Exit Phosphatase Activation in Budding Yeast

**DOI:** 10.1371/journal.pgen.1004907

**Published:** 2015-01-08

**Authors:** Molly Godfrey, Thomas Kuilman, Frank Uhlmann

**Affiliations:** Chromosome Segregation Laboratory, Cancer Research UK London Research Institute, London, United Kingdom; University of California San Francisco, United States of America

## Abstract

Substrate dephosphorylation by the cyclin-dependent kinase (Cdk)-opposing phosphatase, Cdc14, is vital for many events during budding yeast mitotic exit. Cdc14 is sequestered in the nucleolus through inhibitory binding to Net1, from which it is released in anaphase following Net1 phosphorylation. Initial Net1 phosphorylation depends on Cdk itself, in conjunction with proteins of the Cdc14 Early Anaphase Release (FEAR) network. Later on, the Mitotic Exit Network (MEN) signaling cascade maintains Cdc14 release. An important unresolved question is how Cdc14 activity can increase in early anaphase, while Cdk activity, that is required for Net1 phosphorylation, decreases and the MEN is not yet active. Here we show that the nuclear rim protein Nur1 interacts with Net1 and, in its Cdk phosphorylated form, inhibits Cdc14 release. Nur1 is dephosphorylated by Cdc14 in early anaphase, relieving the inhibition and promoting further Cdc14 release. Nur1 dephosphorylation thus describes a positive feedback loop in Cdc14 phosphatase activation during mitotic exit, required for faithful chromosome segregation and completion of the cell division cycle.

## Author Summary

During the cell cycle, a specific sequence of events leads to the formation of two daughter cells from one mother cell. Progression through the cell cycle is tightly controlled, with events occurring in the right place at the right time. Exactly how this is achieved is still being elucidated. In budding yeast, the events occurring during the final cell cycle phase – “mitotic exit” – are controlled by the phosphatase Cdc14. It is kept sequestered and inactive until it is needed for mitotic exit, at which time it is rapidly released. In this study, we have identified a new regulator of Cdc14 activity, the protein Nur1. In a series of experiments, we saw that Nur1 acts both upstream and downstream of Cdc14 activation, thereby creating a positive feedback loop. On the one hand, Nur1 contributes to inhibiting Cdc14 until the start of mitotic exit. On the other hand, through the actions of Cdc14 itself, Nur1 is disabled as an opponent of the phosphatase. This creates a robust system, rapidly switching between two opposing states and thus driving forward the mitotic exit transition.

## Introduction

Cellular reproduction is a highly regulated process that is controlled on a multiplicity of levels, ensuring orderly progression through the different phases of the cell cycle and accurate partitioning of the genome. At the heart of eukaryotic cell cycle control lie cyclin-dependent kinases (Cdks) and their opposing phosphatases [1], [2]. In *Saccharomyces cerevisiae*, the Cdk subunit Cdc28 associates with a series of cell cycle stage-specific cyclins to bring about Cdk activity. It is opposed by the main Cdk-counteracting phosphatase Cdc14, which reverses Cdk phosphorylation events during mitotic exit [3]. The changing balance between Cdk and Cdc14 phosphatase activities at this stage of the cell cycle serves to order mitotic exit events, such as spindle elongation and chromosome segregation followed by spindle disassembly and ultimately cytokinesis [2], [4]. So far, only a few of the Cdk substrates, whose dephosphorylation brings about mitotic exit, have been characterized [5]–[9]. During mitotic exit, Cdc14 is also essential for the downregulation of Cdk activity, on the one hand by promoting mitotic cyclin degradation, via dephosphorylation of the Anaphase Promoting Complex (APC) activator Cdh1, and on the other by promoting accumulation of the Cdk inhibitor Sic1 [3], [10], [11].

Cdc14 activity is stringently regulated. During most cell cycle phases, Cdc14 is sequestered in the nucleolus, and thus inactive, through inhibitory binding to Net1 [12]–[14]. It is thought that Cdc14 release from Net1 occurs following phosphorylation of the latter, which can be achieved by a series of kinases including Cdk, Polo and MEN kinases, thus reducing Net1 affinity for Cdc14 [15]–[17]. Net1 phosphorylation, and thus Cdc14 release, is prevented until early anaphase by the action of the phosphatase PP2A^Cdc55^, which keeps Net1 under-phosphorylated. At anaphase onset, the protease separase is activated after APC-mediated destruction of its inhibitor securin. Separase now cleaves the chromosomal cohesin complex to trigger sister chromatid segregation. At the same time, separase uses a non-proteolytic activity to downregulate PP2A^Cdc55^ [18], [19]. This swings the phosphorylation balance on Net1 towards phosphorylation by mitotic Cdk which, with additional help from components of the FEAR network [17], [20], initiates Cdc14 release.

While Cdc14 release during the early stages of anaphase depends on mitotic Cdk activity [17], [19], mitotic cyclins are being degraded at this time and Cdk activity is in decline. It is thought that declining Cdk and increasing Cdc14 contribute to activation of the MEN, a G-protein coupled signaling cascade consisting of the GTPase Tem1, its regulators Lte1 and Bub2/Bfa1, and its downstream kinases Cdc15 and Dbf2/Mob1 [21]–[24]. Both Cdc15 and Mob1 are Cdk targets and their dephosphorylation in mid anaphase contributes to MEN activation [20], [25], [26]. Active MEN kinases in turn are thought to have the potential to maintain Net1 phosphorylation. However, how Cdc14 release is sustained while Cdk activity declines between anaphase onset and MEN activation has remained poorly understood.

Timely Cdc14 activation in early anaphase is important for successful chromosome segregation. It is required to stabilize the anaphase spindle and is also required for completion of chromosome segregation, in particular telomeres and the late segregating rDNA locus [6], [7], [27], [28]. Cdc14 promotes the condensation and segregation of the repetitive rDNA region during anaphase and *cdc14* mutants display rDNA segregation failure despite unobstructed cohesin cleavage. How Cdc14 promotes rDNA segregation is still being debated. Condensin is recruited to the rDNA in anaphase in a Cdc14-dependent manner, where it appears to promote decatenation of the locus, making the condensin complex a prime candidate for Cdc14 regulation [28]–[30]. It has also been suggested that Cdc14 downregulates rDNA transcription by RNA polymerase I, which could facilitate condensin access to the locus [31], [32]. On the other hand, rRNA synthesis continues unabated during mitotic exit, making this hypothesis appear less likely [33]. In any event, a Cdc14 target that is dephosphorylated to promote rDNA condensation and segregation in anaphase remains unknown.

In this study, we take advantage of our recent phosphoproteome analysis of budding yeast mitotic exit [34]. In the search for Cdc14 targets that have a role in regulating rDNA segregation, we identified the nuclear rim protein Nur1 as a Cdc14 substrate. Failure to dephosphorylate Nur1 causes rDNA missegregation, however, this turns out to be the consequence of compromised Cdc14 activation rather than a specific rDNA segregation defect. This leads us to discover that Nur1 has a previously uncharacterized role in Cdc14 inhibition, and that its inhibitory activity is phosphorylation-dependent. Constitutive Nur1 phosphorylation delays Cdc14 activation, while non-phosphorylatable Nur1 causes premature Cdc14 activation. Thus, Cdc14-dependent Nur1 dephosphorylation in early anaphase forms a positive feedback loop to promote further Cdc14 release, with important implications for faithful chromosome segregation.

## Results

### Nur1 is a Cdc14 target in anaphase

In the search for Cdc14 targets that promote rDNA condensation and segregation during anaphase, we reviewed the phosphoproteome of budding yeast mitotic exit. Cells were arrested in metaphase by depletion of the APC coactivator Cdc20, then synchronous mitotic exit progression was induced by ectopic expression of the Cdc14 phosphatase. Mass spectrometry was used to survey the disappearance of phosphopeptides over the course of mitotic exit, with the original intention to identify proteins whose dephosphorylation controls cytokinesis [34].

**Figure 1:**
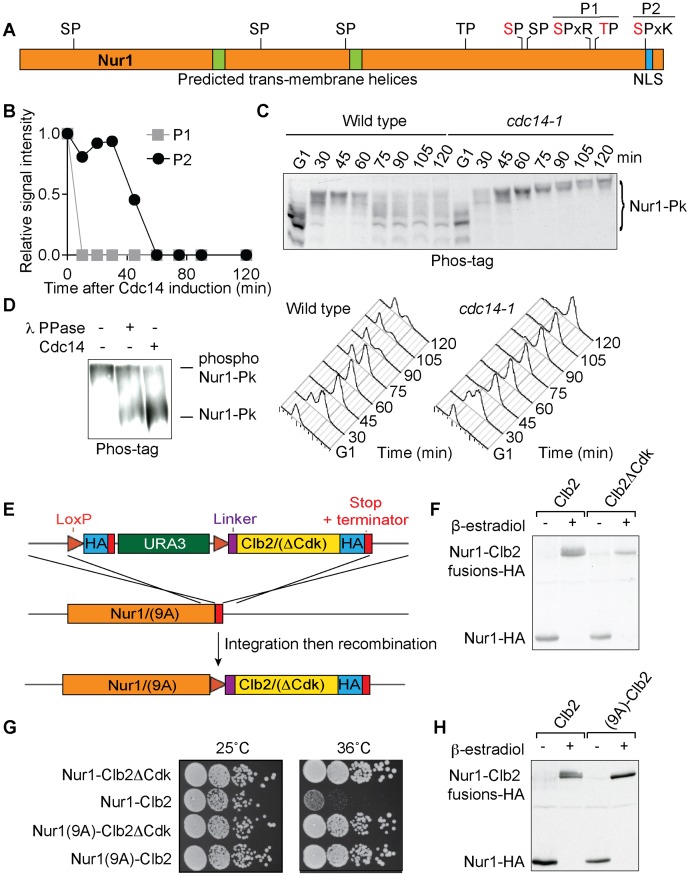
Nur1 dephosphorylation by Cdc14 in anaphase. A . Schematic representation of Nur1. Putative (black) and confirmed (red) Cdk phosphorylation sites, as well as other predicted landmarks, are indicated. P1, SLpSPLRKpTPLSAR; P2, NDINSILRpSPK. **B**. Nur1 phosphopeptide abundance over the course of Cdc14-induced mitotic exit. Compare reference [34] for details. **C**. Nur1 phosphorylation-dependent mobility shifts during synchronous cell cycle progression at 35.5°C, in wild-type and *cdc14-1* cells, were analyzed using Phos-tag gels and Western blotting. Cell cycle progression was monitored by FACS analysis of DNA content. **D**. *In vitro* dephosphorylation of Nur1 by Cdc14. Immunopurified Nur1 was subjected to the indicated phosphatase or control treatments and analyzed by Phos-tag gel electrophoresis. **E**. Design scheme of the Nur1-Clb2 fusion strains. **F**. Western blot demonstrating replacement of endogenous Nur1 with Nur1-Clb2, or Nur1-Clb2ΔCdk, 4 hours after β-estradiol addition. **G**. Serial dilution assay to compare survival of Nur1-Clb2, Nur1-Clb2ΔCdk, Nur1(9A)-Clb2 and Nur1(9A)-Clb2ΔCdk-expressing cells, at 25°C and 36°C. **H**. Western blot demonstrating replacement of Nur1 or Nur1(9A) with Nur1-Clb2 and Nur1(9A)-Clb2, respectively, 4 hours after β-estradiol addition.

Among the proteins that were dephosphorylated in response to Cdc14 expression, in addition to cytokinesis regulators, we identified the nuclear rim protein Nur1 (Fig. 1A, B). Along with its binding partner Src1, Nur1 is involved in tethering rDNA and telomeres to the nuclear envelope, its absence leading to decreased rDNA repeat stability, unequal rDNA segregation, as well as loss of telomere stability and silencing [35]–[37]. Nur1 was previously identified as a Cdk target, containing nine putative Cdk phosphorylation sites, of which four have been confirmed in mass spectrometry studies (Fig. 1A) [34], [38]. Of these four, our phosphoproteome analysis covered three phosphorylation sites on two phosphopeptides. These disappeared with early to intermediate timing, relative to the phosphopeptides of all detected proteins, during Cdc14 induced mitotic exit (Fig. 1B).

To confirm cell cycle-dependent Nur1 phosphorylation, and the role of Cdc14 in its dephosphorylation, we monitored the electrophoretic mobility of Nur1 using Phos-tag gels during synchronous cell cycle progression. Cells were arrested in G1 by pheromone α-factor treatment, before release to progress through the cell cycle at 35.5°C and either rearrest in the next G1 phase by readdition of α-factor, or arrest in late mitosis following Cdc14 inactivation using the temperature sensitive *cdc14-1* allele [39]. Protein extracts were prepared at the indicated times and cell cycle progression was monitored by FACS analysis of DNA content (Fig 1C). Phos-tag gel analysis of the protein extracts revealed the appearance of slower migrating Nur1 isoforms 30 minutes after release from G1 arrest, coincident with the time of S-phase, presumably due to phosphorylation (Fig. 1C). During undisturbed cell cycle progression, Nur1 reached its slowest migration at 45 minutes, in G2/M, before faster migrating forms appeared again at 60–75 minutes. This pattern is consistent with dephosphorylation during early anaphase, before cells completed cytokinesis at 90 minutes. When Cdc14 was inactivated, in the *cdc14-1* strain, dephosphorylation was no longer observed and Nur1 accumulated in slow migrating forms in the late mitotic arrest. To confirm that the mobility shift observed on the Phos-tag gels is indeed due to Nur1 phosphorylation, Nur1 was immunoprecipitated from cells arrested in mitosis by nocodazole treatment and incubated in a control buffer, or in the presence of either λ-phosphatase or purified recombinant Cdc14 [4]. Incubation with either phosphatase, but not control incubation, led to conversion to faster migrating species on the Phos-tag gel, confirming that the slower migrating forms are the consequence of phosphorylation (Fig. 1D).

### Nur1 dephosphorylation by Cdc14 is required for cell survival at higher temperature

In order to examine the importance of Nur1 dephosphorylation, we took advantage of a strategy to create constitutively Cdk phosphorylated proteins by covalent fusion to a mitotic cyclin (Fig. 1E) [34], [40]. In brief, gene targeting was used to fuse the *NUR1* gene at its genomic locus with a mitotic cyclin Clb2 tagging cassette. Clb2 is modified to lack localization and destruction signals, so as not to interfere with Nur1 function. After gene targeting, *NUR1* remains initially separated from the cyclin tag by a selectable marker, flanked by *loxP* recognition sites for the Cre recombinase. The marker is then removed through the action of β-estradiol-activatable Cre-ER recombinase, following hormone addition to the growth medium. As a control, a similar tag was created in which Clb2 harbors three additional point mutations that prevent it from interacting with and recruiting the Cdc28 kinase subunit (denoted Clb2ΔCdk, see Materials and Methods for details). An HA epitope is also included in the tag to facilitate detection, both before and after excision of the marker.

We found there was virtually complete conversion of Nur1 to Nur1-Clb2 and Nur1-Clb2ΔCdk, respectively, four hours following β-estradiol addition (Fig. 1F). Furthermore, Western blotting revealed a haze of slower migrating forms in case of the Nur1-Clb2 fusion protein, but a sharp, faster migrating band in case of Nur1-Clb2ΔCdk, consistent with increased Nur1 phosphorylation due to the Clb2 fusion.

We next examined the effect of the fusions on cell survival. Cells expressing Nur1-Clb2 are viable at 25°C but unable to grow at a higher temperature of 36°C (Fig. 1G). In comparison, Clb2ΔCdk fusion did not affect cell growth at the high temperature, suggesting that temperature sensitivity is caused by the continuous presence of Cdk activity close to Nur1. As the Nur1-Clb2 fusion appears to cause increased Nur1 phosphorylation, constitutive phosphorylation could be the cause of temperature sensitive growth. Alternatively, phosphorylation of proteins in the vicinity of Nur1, due to the increased local Cdk concentration, could cause the temperature sensitivity. To differentiate between these possibilities, we created a version of Nur1, in which its 9 Cdk consensus phosphorylation sites were replaced by alanines (Nur1(9A)). We then repeated the process of generating Nur1(9A)-Clb2 and Nur1(9A)-Clb2ΔCdk fusions. The absence of Cdk phosphorylation sites on Nur1 restored temperature resistant growth following Clb2 fusion (Fig. 1G). It also prevented the Nur1 mobility shift following Clb2 fusion. (Fig. 1H). This suggests that indeed persistent Nur1 phosphorylation on its Cdk phosphorylation sites is the cause for a temperature sensitive growth defect.

### Nur1 dephosphorylation promotes timely rDNA segregation

**Figure 2:**
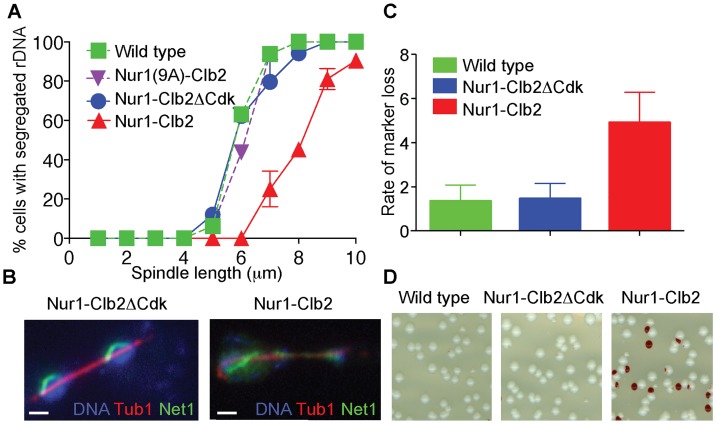
Nur1-Clb2 delays rDNA segregation. **A**. rDNA segregation as a function of spindle length, compared between Nur1-Clb2 and Nur1-Clb2ΔCdk-expressing strains. At least 15, but typically more, cells were scored for each spindle length category. The mean and standard deviation from three independent experiments is shown. Wild type and Nur1(9A)-Cdk strains were also included in one repeat of the experiment. **B**. Representative images of rDNA segregation in a Nur1-Clb2ΔCdk cell, and of an anaphase bridge formed of undercondensed rDNA in a Nur1-Clb2 cell. The rDNA was visualized by the rDNA binding protein Net1, fused to YFP, the spindle was detected with an antibody against α-tubulin, DNA was stained with 4',6-diamidino-2-phenylindole (DAPI). **C**. *ADE2* marker loss from the rDNA repeats is shown as a percentage of half red-sectored colonies after plating the indicated strains, with **D**. A representative field of colonies shown for each genotype. Around 500 colonies were scored for each strain. The mean and standard deviation from four independent experiments is shown.

In order to study the effect of persistent Nur1 phosphorylation on rDNA segregation, we tagged the rDNA binding protein Net1 with YFP to visualize the behavior of the rDNA locus. We synchronized a cell population by α-factor block and release at 36°C and monitored rDNA segregation as cells progressed through mitosis. As an internal marker for segregation timing, we recorded the length of the elongating anaphase spindle in each cell, as well as whether or not the rDNA had separated and segregated into two opposite cell halves. In both a wild type control strain, as well as in the Nur1-Clb2ΔCdk or Nur1(9A)-Clb2 strain, rDNA segregation started at a spindle length of 5–6 µm and was complete by the time spindles reached 8 µm in length. In contrast, in Nur1-Clb2 cells, rDNA segregation only started at spindle lengths of 7–8 µm and never reached completion even when spindles were fully elongated (Fig. 2A). Cytological observation of the rDNA locus showed that it reached its expected tightly condensed state in opposite cell halves in wild type and Nur1-Clb2ΔCdk cells (Fig. 2B). In contrast, the rDNA often appeared stretched and uncondensed in Nur1-Clb2 cells. These observations are consistent with the possibility that Nur1 dephosphorylation promotes rDNA condensation, which in turn is required for its timely segregation. Notably, an rDNA segregation defect of similar extent to that in Nur1-Clb2 cells is seen after inactivation of the chromosomal condensin complex [27]–[30]. An rDNA segregation defect was also observed, albeit less pronounced, in Nur1-Clb2 cells at 25°C (S1 Fig.). Taken together, we conclude that constitutive phosphorylation of Nur1 leads to delayed rDNA segregation, coincident with defective rDNA condensation.

A consequence of rDNA segregation defects is unequal sister chromatid exchange and consequent chromosomal instability. As a measure for rDNA stability, we assessed the loss rate of an *ADE2* marker within the rDNA repeats [36], [41]. *ADE2* loss causes accumulation of a red intermediate metabolite in the adenine biosynthesis pathway, thus causing the colony color to turn red. *ADE2* loss during the first cell division after plating on agar medium will generate half red-sectored colonies. We therefore counted the fraction of half red-sectored colonies, among all colonies, as a measure for the *ADE2* loss rate from the rDNA. The loss rate was approximately five-fold elevated in the Nur1-Clb2 strain at 25°C, compared to a wild type and Nur1-Clb2ΔCdk controls (Fig. 2C). This is indicative of rDNA instability, probably as the consequence of rDNA segregation defects caused by the Nur1-Clb2 fusion, even at a permissive temperature.

### Persitent Nur1 phosphorylation delays mitotic progression

**Figure 3:**
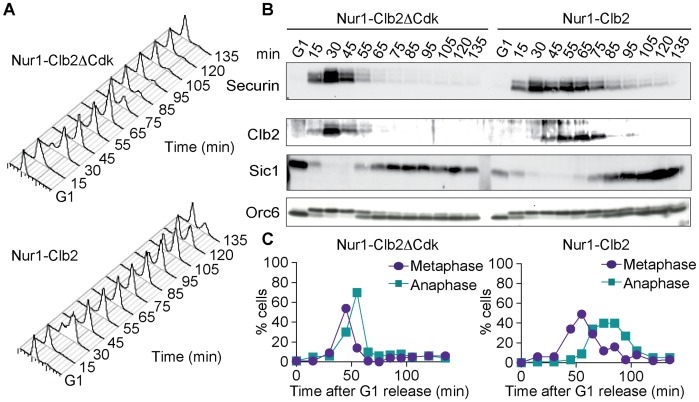
Nur1-Clb2 causes a mitotic exit defect. Nur1-Clb2 and Nur1-Clb2ΔCdk cells were synchronized in G1 by α-factor treatment and released to progress through the cell cycle at 36°C, before being rearrested in the following G1. At time points throughout the cell cycle we monitored, **A**. Cell cycle progression by FACS analysis of DNA content. **B**. Western blot analysis of the cell cycle markers securin, Clb2, Sic1 and Orc6, and **C**. The percentages of cells displaying metaphase (1–3 µm) or anaphase (>3 µm) spindles were scored. 100 cells were counted at each time point.

Nur1 is a nuclear rim protein that makes contact with the rDNA, so its phosphorylation status could directly affect rDNA condensation. Alternatively, Nur1 phosphorylation could indirectly affect rDNA condensation and segregation. In particular, delayed rDNA segregation is a hallmark of mitotic exit defects, as for instance observed in FEAR pathway mutants. In order to differentiate between these possibilities, we monitored cell cycle progression of Nur1-Clb2ΔCdk and Nur1-Clb2 cells. Cells were synchronized in G1 by α-factor treatment, released to pass through a synchronous cell cycle at 36°C, before being rearrested in the following G1 by α-factor readdition. FACS analysis of DNA content showed that Nur1-Clb2 cells spent at least 20 minutes longer with a 2C DNA content (Fig. 3A), i.e. show delayed cell cycle progression between G2 and cytokinesis, compared to the control.

To delineate where Nur1-Clb2 cells are delayed in cell cycle progression, we analyzed several cell cycle markers using Western blotting at frequent time intervals during the time course (Fig. 3B). Appearance of securin and Clb2 at around the time of S-phase was indistinguishable between the control and Nur1-Clb2 cells, as was phosphorylation of Orc6 that takes place at that time. However, destruction of both securin and Clb2 were markedly delayed in Nur1-Clb2 cells, as was Orc6 dephosphorylation during mitotic exit and reaccumulation of the Cdk inhibitor Sic1. This pattern suggests a delay of Nur1-Clb2 cells during mitosis and mitotic exit. To distinguish whether the mitotic delay takes place before or after the metaphase to anaphase transition, we monitored spindle morphology during cell cycle progression (Fig. 3C). This revealed that Nur1-Clb2 cells persisted for somewhat longer with short metaphase spindles (1–3 µm in length), as well as a pronounced prolongation of the time that cell persisted with long anaphase spindles (>3 µm in length). These observations are consistent with a delay of Nur1-Clb2 cells both at the metaphase to anaphase transition as well as during mitotic exit. A similar delay in mitotic progression, albeit less pronounced, was observed in Nur1-Clb2 cells at 25°C (S1 Fig.). To confirm that the mitotic delay due to Nur1-Clb2 was caused by persistent Nur1 phosphorylation, we used cells harboring Nur1(9A). Clb2 fusion to Nur1(9A) no longer delayed cell cycle progression (S2 Fig.), indicating that indeed persistent Nur1 phosphorylation slows down mitotic progression. These findings further open the possibility that rDNA condensation and segregation defects seen in Nur1-Clb2 cells are a consequence of a mitotic exit delay.

### Nur1-Clb2 delays Cdc14 release

**Figure 4:**
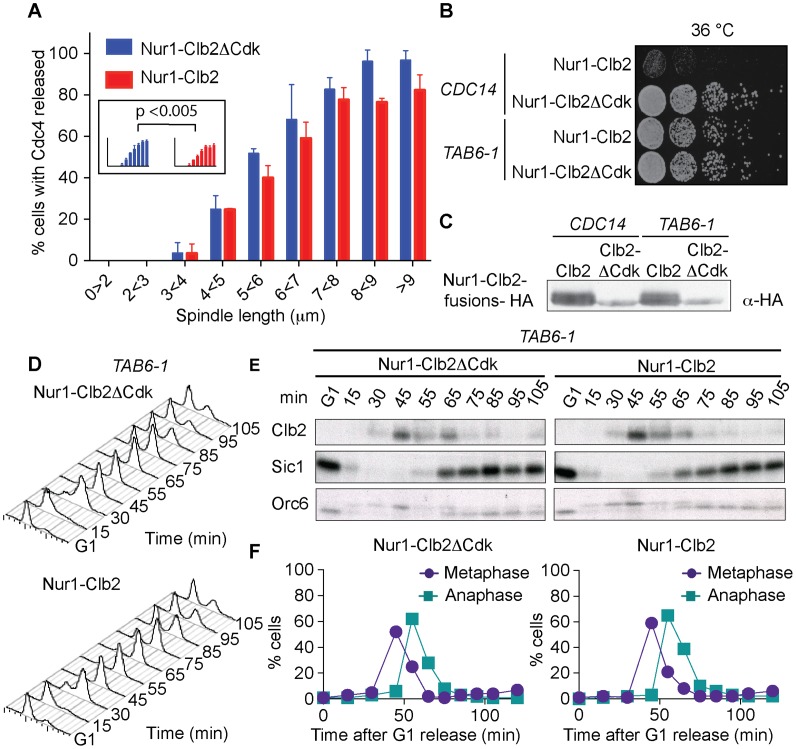
Nur1-Clb2 delays Cdc14 release. **A**. Quantification of Cdc14 release, in experiments performed as in Fig. 3, relative to spindle length. At least 15, but typically more, cells were counted for each spindle length category. The mean and standard deviation from 3 independent experiments is shown. A logistic regression analysis showed that Cdc14 release timing in the Nur1-Clb2 strain was significantly delayed. **B**. Serial dilution assay demonstrating rescue of cell survival of Nur1-Clb2 cells by dominant active Cdc14^TAB6-1^. **C**. Western blot samples taken of the cells in **B**, before they were plated, shows that Cdc14^TAB6-1^ did not alter the Nur1-Clb2 phosphorylation status. **D.-F.** Active Cdc14 rescues mitotic progression of Nur1-Clb2 cells. As Fig. 3A-C, but strains carried the *CDC14^TAB6-1^* allele.

The mitotic delay observed in Nur1-Clb2 cells is reminiscent of that seen in cells with an inactive FEAR pathway [42]. In those cells, delayed Cdc14 phosphatase activation slows mitotic exit progression and rDNA segregation [27], [28]. Given the phenotypic similarities, we examined the timing of Cdc14 release in Nur1-Clb2, compared to wild type and Nur1-Clb2ΔCdk, cells. Again, we synchronized cells in G1 and then measured the timing of Cdc14 release as a function of spindle length as cells passed through mitosis. In several repeats of this experiment, we detected a small but statistically significant delay to Cdc14 release in the Nur1-Clb2 strain (Fig. 4A). This suggests that phosphorylated Nur1 impedes Cdc14 activation, a likely cause for delayed mitotic exit and rDNA segregation defects.

To investigate whether delayed Cdc14 activation is indeed the cause for mitotic defects in Nur1-Clb2 cells, we tested whether the dominant active *CDC14^TAB6-1^* allele, which leads to Cdc14 being less tightly bound by Net1 [43], restores cell survival at high temperature. Indeed, we found that *CDC14^TAB6-1^* almost completely restored temperature-resistant growth of the Nur1-Clb2 strain (Fig. 4B). As a control, we confirmed that *CDC14^TAB6-1^* did not cause dephosphorylation of Nur1-Clb2, e.g. due to the presence of higher than normal levels of Cdc14 activity. Western blotting revealed that Nur1-Clb2 mobility, in particular its slower migrating forms, remained unaffected by *CDC14^TAB6-1^* (Fig. 4C). This confirms that the temperature sensitive growth due to persistent Cdk phosphorylation of Nur1 is caused by defective Cdc14 activation.

We also monitored the dynamics of cell cycle progression in the Nur1-Clb2 strain rescued by the *CDC14^TAB6-1^* allele. This revealed that the mitotic delay caused by Nur1-Clb2 is largely reduced in cells carrying the *CDC14^TAB6-1^* allele (Fig. 4D). Both the delays at the metaphase to anaphase transition, as well as the delay during mitotic exit, were ameliorated. FACS analysis of DNA content as well as Western blotting analysis of Clb2 levels and of Orc6 dephosphorylation during a synchronous cell cycle confirmed that Nur1-Clb2 no longer affects mitotic progression in *CDC14^TAB6-1^* cells. This indicates that the mitotic defects and associated loss of viability at high temperature in Nur1-Clb2 cells are caused by defective Cdc14 activation.

### Phospho-Nur1 counteracts Cdc14 release in early anaphase

**Figure 5:**
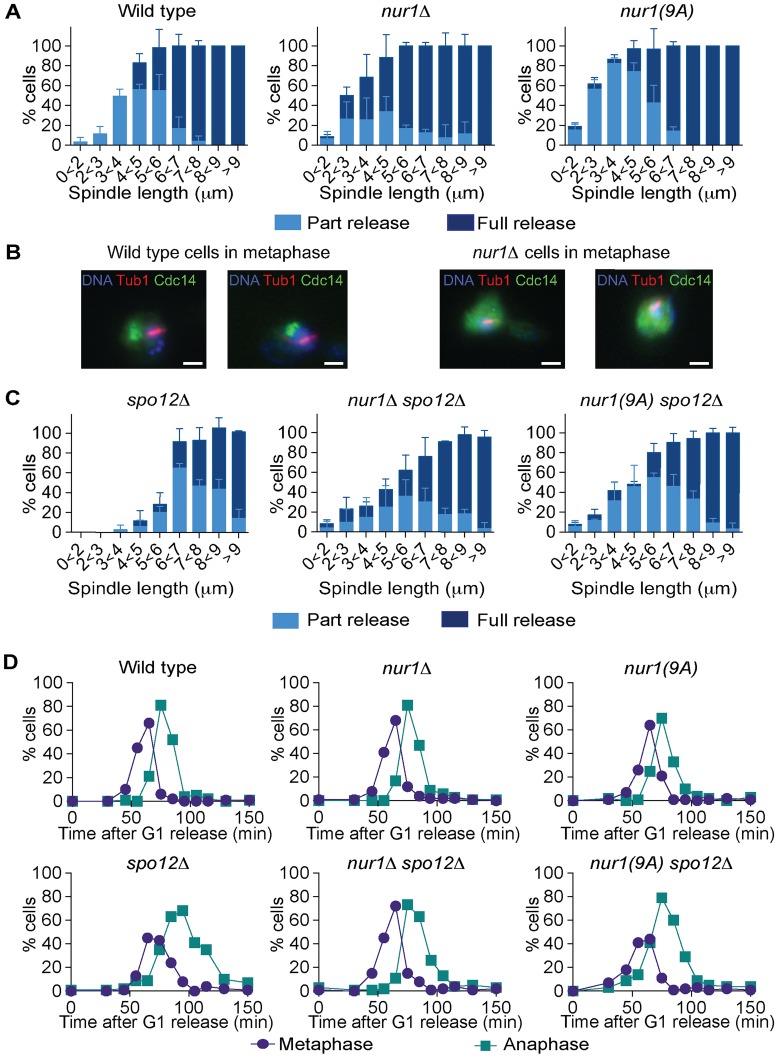
Phosphorylated Nur1 contributes to Cdc14 sequestration in early anaphase. **A**. Quantification of Cdc14 release versus spindle length, as in Fig. 4A, in wild type, *nur1Δ* and *nur1(9A)* cells. Cdc14 release was subdivided into ‘Partial release’, when Cdc14 was detectably released into the nucleus but some nucleolar enrichment persisted and ‘Full release’, when no nucleolar Cdc14 enrichment remained detectable. The mean and standard deviation from three independent experiments is shown. **B**. Images of Cdc14 in wild type and *nur1Δ* cells. The metaphase state is confirmed by the presence of a short spindle, stained with an α-tubulin antibody. Cdc14-GFP was detected with an α-GFP antibody, DNA was counterstained with DAPI. **C**. As **A**, but cells carried compromised FEAR network activity due to the *spo12Δ* allele. **D**. Progression through mitosis of the strains above was measured by counting percentages of cells displaying metaphase (1–3 µm) or anaphase (>3 µm) spindles during synchronous cell cycle progression.

If Nur1, especially the Cdk phosphorylated form, prevents Cdc14 release in early anaphase, then eliminating Nur1 or removing its Cdk phosphorylation sites, should facilitate Cdc14 release. To investigate this possibility, we compared Cdc14 release kinetics in synchronized populations of wild type, *nur1Δ* and *nur1(9A)* cells. Strikingly, Cdc14 was released in over half of *nur1Δ* and *nur1(9A)* cells in early anaphase at spindle lengths below 3 µm, when Cdc14 release is almost never seen in wild type cells (Fig. 5A). Even in metaphase cells with short (1-2 µm) spindles, when Cdc14 is normally tightly sequestered, a substantial fraction of *nur1Δ* and *nur1(9A)* cells displayed released Cdc14 (Fig. 5B). This suggests that phosphorylated Nur1 restricts Cdc14 release in early anaphase.

Cdc14 activation is promoted by the FEAR network in early anaphase. To further study the impact of Nur1 on Cdc14 release at this time, we introduced the *nur1Δ* and *nur1(9A)* alleles into a *spo12Δ* strain, lacking a key component of the FEAR network [20]. In the *spo12Δ* background, Cdc14 release is delayed until spindles reach about 6 µm in length. Deletion of *nur1*, or its replacement with *nur1(9A)*, restored Cdc14 early anaphase release (Fig. 5C). The Cdc14 release profile in the *nur1Δ spo12Δ* and *nur1(9A) spo12Δ* double mutant strains approached that of wild type cells. However, at short spindle lengths, *nur1Δ spo12Δ* and *nur1(9A) spo12Δ* cells still showed premature Cdc14 release, as compared to wild type, while at longer spindle lengths the rescue did not fully match wild type release levels. These findings confirm that phosphorylated Nur1 is a potent inhibitor of Cdc14 release in early anaphase and that the FEAR network acts to overcome Cdc14 inhibition by Nur1. However, the incomplete rescue of Cdc14 release in *spo12Δ* cells by Nur1 ablation suggests that the FEAR network acts at least in part by a mechanism different from inactivating Nur1.

We next compared the kinetics of mitotic progression between wild type, *nur1Δ* and *nur1(9A)* strains. Despite the advanced Cdc14 release in the latter strains, the timing of progression through mitosis, as measured by the fractions of cells displaying metaphase and anaphase spindles at each time point, was indistinguishable from wild type (Fig. 5D). In a *spo12Δ* background, cells showed approximately a 20 minute delay in anaphase. In this case, *nur1Δ* or *nur1(9A)* restored the kinetics of mitotic progression close to wild type (Fig. 5D). This confirms that Nur1, specifically its phosphorylated form, can delay mitotic progression.

### Nur1 inactivation does not compensate for MEN defects

**Figure 6:**
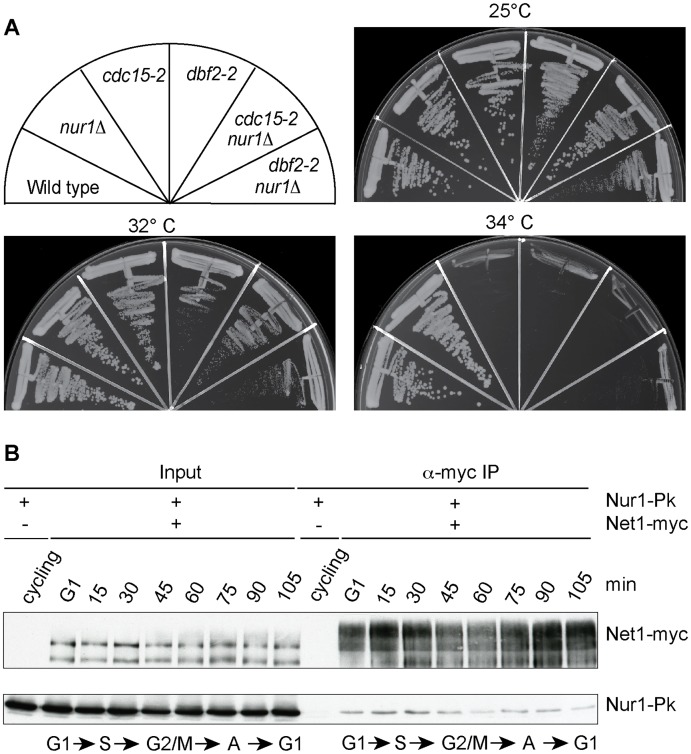
Nur1 acts in early anaphase and interacts with Net1. **A**. Nur1 deletion does not reduce the requirement for the MEN. Strains of the indicated genotypes were streaked on YPD agar plates and grown at the temperatures shown. **B**. Nur1 interacts with Net1 throughout the cell cycle. Cell extracts were prepared from aliquots of a culture passing through a synchronous cell cycle at the indicated times. Co-immunoprecipitation of Nur1-Pk with Net1-myc was examined. A strain expressing Nur1-Pk but lacking the Net1-myc epitope served as a control. Cell cycle progression was monitored by FACS analysis of DNA content, the prevalent cell cycle stages are indicated.

Inactivation of Nur1, by deletion or mutation of its Cdk phosphorylation sites, augments Cdc14 release in early anaphase and almost completely compensates for loss of FEAR network components. This could be because phospho-regulation of Nur1 has a role specific to early anaphase. Alternatively, Nur1 might be a general Cdc14 inhibitor at all stages of mitotic exit. To differentiate between these two possibilities, we tested whether Nur1 inactivation can also compensate for partial loss of MEN signaling. We used conditional thermosensitive alleles in two MEN kinases *cdc15-2* and *dbf2-2* and asked whether *nur1* deletion would improve cell growth at a semi-permissive temperature when MEN signaling is partially compromised. However, *cdc15-2 nur1Δ* and *dbf2-2 nur1Δ* double mutants lost viability at increasing temperatures in a fashion indistinguishable from the parental *cdc15-2* and *dbf2-2* strains (Fig. 6A). Thus, Nur1 does not appear to act later during mitotic exit, when the MEN takes control of Cdc14 release. Instead, Nur1 function as a Cdc14 inhibitor appears to be restricted to early anaphase. This conclusion is consistent with the Nur1 phosphorylation pattern during mitotic exit. Nur1 is Cdk phosphorylated during early mitosis, when it counteracts Cdc14. In anaphase, Nur1 becomes dephosphorylated due to Cdc14 action and thus looses its inhibitory effect on Cdc14.

### Nur1 is a Net1-binding protein

To investigate the mechanism of how Nur1 counteracts Cdc14, we asked whether Nur1 is physically linked to components of mitotic exit control. Affinity purified fractions of Nur1, analyzed by sensitive mixture mass spectrometry, contained Heh1 and mitotic monopolin, as well as a Net1 peptide [36]. However, while mitotic monopolin resides in the nucleolus, Nur1 could not be detected on the rDNA together with Net1 by chromatin immunoprecipitation [36], [44]. To clarify whether Nur1 interacts with Net1, we performed a co-immunoprecipitation experiment. A Net1-myc strain was synchronized in G1 and cell extracts prepared at regular time intervals following release into synchronous cell cycle progression. Net1 was immunoprecipitated from the extracts using an α-myc antibody. Nur1 coprecipitated with Net1 at all stages of the cell cycle, with little fluctuation to the efficiency of the interaction (Fig. 6B). No Nur1 was recovered in a parallel control immunoprecipitation from extracts of a strain lacking the Net1 myc epitope. In addition, we detected Cdc14 in immunoprecipitates of Nur1 throughout the cell cycle. While we do not currently know with which of Net1 and/or Cdc14 Nur1 makes direct contact, this finding opens the possibility that Nur1 directly influences Cdc14 inhibition in conjunction with Net1.

A key event during Cdc14 activation in early anaphase is Cdk phosphorylation of Net1 on at least six Cdk consensus phosphorylation sites. It could therefore be that Nur1 antagonizes Cdc14 by counteracting Net1 phosphorylation. As a start to investigate this, we took advantage of the *net1-6Cdk* allele that lacks these six Cdk phosphorylation sites and, as a consequence, delays Cdc14 activation and causes a short mitotic exit delay [17]. If Nur1 impacts on Cdc14 by counteracting Net1 phosphorylation, then Nur1 inactivation will not be able to correct the mitotic exit delay of *net1-6Cdk* cells. In contrast, if Nur1 counteracts Cdc14 in a pathway different from Net1 phosphorylation, then its deletion should be able to advance Cdc14 activation in *net1-6Cdk* cells, just as it did in the *spo12*Δ background. However, *nur1* deletion did not improve Cdc14 release nor reduce the mitotic exit delay of *net1-6Cdk* cells (S3 Fig.). This suggests that Nur1 contributes to Cdc14 regulation most likely by influencing Cdk phosphorylation of Net1. It will be an important task to directly study the influence of Nur1 on Net1 phosphorylation.

## Discussion

### Nur1, a new player in budding yeast mitotic exit

The paramount importance of the Cdc14 phosphatase during budding yeast mitotic exit is well established, with the list of its targets and functions increasing. For instance, the incompletely understood role of Cdc14 in cytokinesis has recently come into focus [34], [45], [46]. Concomitantly, our understanding of the regulation of nucleolar release and activation of this phosphatase is deepening [17], [19], [47]–[50].

In this study, we set out to further our molecular knowledge of the role of Cdc14 in rDNA condensation and segregation. A candidate Cdc14 target with potential to impact on rDNA segregation was identified as the nuclear rim protein Nur1 in our recent phosphoproteome screen of budding yeast mitotic exit. Nur1 has been previously linked to regulating rDNA stability. We confirmed Nur1 as Cdc14 substrate *in vivo* and *in vitro*, that is dephosphorylated in early anaphase. To study the role of Nur1 dephosphorylation, we created a constitutively phosphorylated form of Nur1, by fusing it to a cyclin Clb2 moiety. The fusion led to an rDNA segregation delay and reduced viability, which was dependent on the Cdk phosphorylation sites, especially at high temperatures. This confirms Nur1 as a Cdc14 target whose dephosphorylation is critical for successful mitotic exit progression.

**Figure 7:**
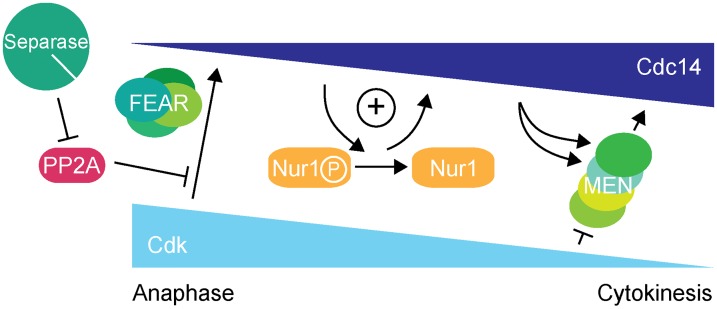
Nur1 establishes a positive feedback loop to promote Cdc14 release in early anaphase. At the metaphase to anaphase transition, Cdc14 starts to be released following APC-mediated securin destruction, which liberates separase to downregulate PP2A^Cdc55^. This allows Cdk to phosphorylate Net1 to initiate Cdc14 release. At the same time Cdk activity is in decline, thus removing the original impetus for Cdc14 release. To counteract this, Nur1 is dephosphorylated by early released Cdc14. This turns off Nur1's ability to inhibit Cdc14 and thus helps to sustain and augment Cdc14 release until the MEN pathway becomes active to maintain Cdc14 release while Cdk activity is further downregulated.

Further investigation revealed that the overt rDNA segregation defect in Nur1-Clb2 cells was likely a secondary consequence of a primary defect in Cdc14 activation. While the Cdc14 substrate(s) that govern rDNA condensation, resolution and segregation in anaphase therefore remain elusive, we have uncovered a previously unknown Cdc14 substrate that acts to sustain Cdc14 activation, thus creating positive feedback of Cdc14 release in early anaphase (Fig. 7).

While inhibition of Cdc14 release by Nur1 is normally restricted to early anaphase while Nur1 is phosphorylated, our Nur1-Clb2 fusion likely extends that period, potentially throughout mitotic exit. Nur1-Clb2 may thus elicit phenotypic consequences beyond the delayed Cdc14 release characteristic of FEAR network mutants. An example of a difference is the temperature sensitive growth of Nur1-Clb2 cells, which is not typically shared by FEAR network mutants. A higher temperature causes cells to progress faster through the cell cycle, presumably rendering them less tolerant to delays or alterations to cell cycle signaling pathways. Phenotypes of late mitotic exit mutants, in particular those involved in cytokinesis, are known to be exacerbated at high temperatures [34], consistent with an impact of Nur1-Clb2 at later stages.

Intriguingly, defective Cdc14 activation in Nur1-Clb2 cells caused not only a mitotic exit delay, but also a delay at the metaphase to anaphase transition. A small amount of Cdc14 is likely to be active by the time of metaphase, when it contributes to dephosphorylation of proteins like Dsn1 and possibly Spo12 [49], [51]. Furthermore, securin dephosphorylation by Cdc14 has been suggested to sharpen the timing of anaphase [52]. Nur1-dependence of regulating these earliest Cdc14 targets might play a role at the metaphase to anaphase transition. We cannot exclude that Nur1 performs additional functions at this time, that might be affected by Nur1-Clb2 fusion and that might contribute to explaining the lethality of Nur1-Clb2 fusion strains at high temperatures.

### A relationship between Nur1 in cell cycle regulation and genome stability?

Our results demonstrate that Nur1, through regulating Cdc14, plays an important role in promoting the accurate timing of mitotic exit events. Nur1 was previously characterized as chromosome linkage protein, connecting chromatin and the inner nuclear membrane, with functions in the maintenance of genome stability and replicative life span [36], [37]. Are these independent functions of this protein, or are they related?

Constitutive Nur1 phosphorylation, caused by the Nur1-Clb2 fusion, increased marker loss at the rDNA, likely due to unequal sister chromatid exchange. A similar level of marker loss at the rDNA was previously reported in *nur1Δ* cells [36]. It will be therefore interesting to examine whether Nur1's role in tethering the rDNA to the nuclear envelope is regulated by its phosphorylation status. It is as yet unknown whether rDNA tethering is modulated during the cell cycle, for instance during rDNA condensation and segregation, and how the rDNA moves relative to the nuclear envelope during anaphase. It has been speculated that cell cycle-dependent Nur1 phosphorylation within a putative nuclear localization signal might affect the nucleocytoplasmic shuttling of the protein, with possible implications both for Cdc14 regulation and rDNA tethering [37]. Our initial observations of Nur1 localization revealed faint Nur1 enrichment at the nuclear envelope, consistent with previous reports [36], [53]. This localization did not noticeably change during cell cycle progression and was not altered as consequence of Clb2 fusion or Cdk phosphosite mutations. Nevertheless, the possibility of Nur1 regulation by localization merits further investigation.

Nur1 interacts with Net1 and, in addition to Net1's role as a Cdc14 inhibitor, one of the phenotypes reported for *net1-1* mutants is a high rate of chromosome loss [54]. This indicates that Net1 plays a role in accurate chromosome segregation that might go beyond its role as cell cycle regulator. The relationships between Net1, Nur1, the phosphoregulation of both proteins and their roles in Cdc14 phosphatase regulation and genome stability are an important topic for further studies.

### Nur1 adds positive feedback to Cdc14 release in early anaphase

Removing Nur1 (*nur1Δ*), or its Cdk phosphorylation sites (*nur1(9A)*), resulted in premature Cdc14 release. Phosphorylated Nur1 therefore fulfills a role in attenuating Cdc14 activation in early anaphase. In a quantitative model in which Cdc14 substrate dephosphorylation timing is determined by the ratio of phosphatase activity versus Cdk kinase activity [2], [4], phospho-Nur1 ensures that Cdc14 activity is kept low and only its earliest targets are dephosphorylated in early anaphase.

While preventing Nur1 phosphorylation causes premature Cdc14 activation, maintaining persistent Nur1 phosphorylation delays Cdc14 release and mitotic exit. Nur1 dephosphorylation by Cdc14 in early anaphase thus engages a positive feedback loop in which phospho-Nur1 as a Cdc14 inhibitor is removed by the very action of Cdc14 itself (Fig. 7). Whether Nur1 dephosphorylation actually stimulates release of Cdc14, or just stops Nur1 from counteracting it, is still an open question. In either case, failure to turn off phospho-Nur1 results in a mitotic exit delay, compromised rDNA segregation and inability to survive at higher temperature. Thus, Nur1 dephosphorylation plays an important role in shaping the Cdc14 activation pattern in early anaphase, until the MEN takes over to sustain Cdc14 release. Additional players might contribute to Cdc14 feedback regulation, including Tof2 [50] or Spo12 [49].

Although we have found that Nur1 interacts with Net1 and Cdc14, the molecular mechanism by which Nur1 counteracts Cdc14 release is not yet understood. The fact that Nur1 no longer influences Cdc14 activation in a *net1-6Cdk* strain suggest that Nur1 acts at the level of Cdk-dependent Net1 phosphorylation. A possible mechanism that is consistent with this observation takes into consideration that Net1 itself is a Cdc14 target *in vivo* and *in vitro* [12], [25]. Cdc14 could prevent its own release if it were allowed access to Cdk phosphorylation sites on Net1. A scenario can be envisaged in which phospho-Nur1 promotes dephosphorylation of Net1 by Cdc14. The required change in accessibility of Net1 phosphorylation sites could be accomplished through conformational changes within a Net1/Nur1/Cdc14 protein complex, depending on the Nur1 phosphorylation state.

In summary, we describe a positive feedback loop, in which Nur1 attenuates Cdc14 release until Nur1 itself is dephosphorylated by Cdc14. This feedback imposes a requirement for the FEAR network to initiate Cdc14 release. Once Cdc14 becomes active, Nur1 dephosphorylation helps to sustain Cdc14 release while Cdk activity declines, until the MEN pathway takes over. In this model, Nur1 can be seen as a bridge between the FEAR network and the MEN signaling cascade.

## Methods and Materials

### Yeast strains and techniques

All strains were of the W303 background and are listed in S1 Table. A strain containing *ADE2* integrated within the rDNA repeats was a kind gift from M. Kaeberlein [41]. Epitope tagging of endogenous genes and gene deletions were performed by gene targeting using polymerase chain reaction (PCR) products [55], [56]. The *nur1(9A)* mutant was engineered by endogenous gene replacement using an integrative plasmid, based on a synthetic DNA construct (GeneArt, Life Technologies). The conditional Nur1-Clb2 and Nur1-Clb2ΔCdk fusions were created as described [34]. In brief, Clb2 lacking its destruction and KEN-boxes [57], as well as its nuclear localization sequence [58], was fused to Nur1, separated by an unstructured 10-mer GGSGTGGSGT linker. In addition, the Clb2ΔCdk mutant contained further 3 point mutations that prevent it from interacting with the Cdc28 kinase subunit, as described [59]. Strains harboring the conditional Clb2-fusion cassettes were grown on medium lacking uracil to maintain the selectable marker and to prevent spontaneous recombination. Marker loop-out was then induced by β-estradiol-dependent activation of Cre recombinase fused to an estradiol binding domain (Cre-EBD78; [60]), by addition of 1 µM β-estradiol to the growth medium.

Yeast cultures were grown in rich YP medium supplemented with 2% glucose [61]. Cell synchronization using α-factor was as described [62]. For cell synchronization at higher temperatures, using the *cdc14-1* or *NUR1-CLB2* backgrounds, cultures were shifted to the higher temperatures at the time of release from α-factor arrest.

### Western blotting, immunoprecipitation and phosphatase assay

Protein extracts for Western blotting were prepared following cell fixation using trichloroacetic acid, as described [63], and analyzed by SDS-polyacrylamide gel electrophoresis (SDS-PAGE). Antibodies used for Western detection were, α-Clb2 (Santa Cruz, sc9071), α-Orc6 (clone SB49); α-Sic1 (Santa Cruz, sc50441), α-Tub1 (clone YOL1/34, AbD Serotec), α-HA (clone 12CA5), α-myc (clone 9E10), α-Pk (clone SV5-Pk1, AbD Serotec). Phos-tag was purchased from Wako Chemicals and added to SDS-polyacrylamide gels along with MnCl_2_ according to the manufacturer's instructions.

For immunoprecipitation, cell extracts were prepared in EBXG buffer (50 mM HEPES pH 8.0, 100 mM KCl, 2.5 mM MgCl_2_, 10% glycerol, 0.25% Triton X-100, 1 mM DTT, protease inhibitors) using glass bead breakage in a Multi Bead Shocker (Yasui Kikai). Extracts were precleared, incubated with antibody and finally adsorbed to Protein A Dynabeads. Beads were washed and elution was carried out in SDS-PAGE loading buffer. For the *in vitro* Nur1 dephosphorylation assay, immunoprecipitation was performed as above, then beads were resuspended in phosphatase buffer and 1 µg λ phosphatase (New England Biolabs), or 8 µg purified recombinant Cdc14 [4], were added, followed by incubation at 30°C for 30 minutes before the reaction was stopped and proteins eluted by addition of SDS-PAGE loading buffer.

### Immunofluorescence microscopy

Indirect immunofluorescence was performed on formaldehyde-fixed cells using the following antibodies, α-GFP, (clone TP401, Torrey Pines Biolabs or ab6556, Abcam), α-Tub1 (clone YOL1/34, AbD Serotec) and FITC and Cy3-dye labeled secondary antibodies (Sigma and Chemicon, respectively). Cells were counterstained with the DNA binding dye 4',6-diamidino-2-phenylindole (DAPI). Fluorescent images were acquired using an Axioplan 2 imaging microscope (Zeiss) equipped with a 100x (NA = 1.45) Plan-Neofluar objective and an ORCA-ER camera (Hamamatsu). Spindle length measurements were carried out in ImageJ.

## Supporting Information

S1 Fig An rDNA segregation and mitotic exit delay in Nur1-Clb2 cells at a permissive temperature of 25°C. **A**. rDNA segregation is delayed in Nur1-Clb2, as compared to Nur1-Clb2ΔCdk cells. Completion of rDNA segregation is plotted as a function of spindle length. **B**. Progression through mitosis is delayed in Nur1-Clb2 cells, as seen by FACS analysis of DNA content of cultures passing through a synchronous cell cycle following α-factor arrest and release at 25°C. **C**. Cells from the timecourse shown in **B** were processed to visualize spindle microtubules by indirect immunofluorescence using an antibody against α-tubulin. A mitotic exit delay in Nur1-Clb2 cells is manifest by the longer persistence of cells containing elongated anaphase spindles.
(TIF)

S2 Fig Nur1 phosphorylation is responsible for the mitotic exit delay seen in Nur1-Clb2 cells. Nur1-Clb2 and Nur1(9A)-Clb2 cells were synchronized in G1 by α-factor treatment and released to progress through the cell cycle at 36°C, before being rearrested in the following G1. At time points throughout the cell cycle we monitored, **A**. cell cycle progression by FACS analysis of DNA content. **B**. levels of the cell cycle markers Clb2, Sic1 and Orc6 by Western blot analysis, and **C**. the percentages of cells displaying metaphase (1–3 µm) or anaphase (>3 µm) spindles. 100 cells were counted at each time point.
(TIF)

S3 Fig Nur1's effect on Cdc14 is dependent on Net1's phosphorylation status. **A**. Progression through mitosis of wild type, *nur1Δ, net1-6cdk* and *nur1Δ net1-6cdk* cells was measured by counting percentages of cells displaying metaphase (1–3 µm) or anaphase (>3 µm) spindles during synchronous cell cycle progression following α-factor arrest and release. **B**. Quantification of Cdc14 release versus spindle length, as in Figure 5, during the experiment above.
(TIF)

S1 Table List of yeast strains used in this study.
(DOCX)

## References

[1] Morgan D (2007) The cell cycle: Principles of control. London: New Science Press.

[2] Uhlmann F, Bouchoux C, López-Avilés S (2011) A quantitative model for cyclin-dependent kinase control of the cell cycle: revisited. Phil Trans R Soc B 366: 3572–3583. 22084384 10.1098/rstb.2011.0082 PMC3203462

[3] Visintin R, Craig K, Hwang ES, Prinz S, Tyers M, et al (1998) The phosphatase Cdc14 triggers mitotic exit by reversal of Cdk-dependent phosphorylation. Mol Cell 2: 709–718. 9885559 10.1016/s1097-2765(00)80286-5

[4] Bouchoux C, Uhlmann F (2011) A quantitative model for ordered Cdk substrate dephosphorylation during mitotic exit. Cell 147: 803–814. 22078879 10.1016/j.cell.2011.09.047

[5] Pereira G, Schiebel E (2003) Separase regulates INCENP-Aurora B anaphase spindle function through Cdc14. Science 302: 2120–2124. 14605209 10.1126/science.1091936

[6] Higuchi T, Uhlmann F (2005) Stabilization of microtubule dynamics at anaphase onset promotes chromosome segregation. Nature 433: 171–176. 15650742 10.1038/nature03240 PMC2586334

[7] Woodbury EL, Morgan DO (2007) Cdk and APC activities limit the spindle-stabilizing function of Fin1 to anaphase. Nat Cell Biol 9: 106–112. 17173039 10.1038/ncb1523

[8] Khmelinskii A, Roostalu J, Roque H, Antony C, Schiebel E (2009) Phosphorylation-dependent protein interactions at the spindle midzone mediate cell cycle regulation of spindle elongation. Dev Cell 17: 244–256. 19686685 10.1016/j.devcel.2009.06.011

[9] Mirchenko L, Uhlmann F (2010) Sli15^INCENP^ dephosphorylation prevents mitotic checkpoint reengagement due to loss of tension at anaphase onset. Curr Biol 20: 1396–1401. 20619650 10.1016/j.cub.2010.06.023 PMC2964898

[10] Zachariae W, Schwab M, Nasmyth K, Seufert W (1998) Control of cyclin ubiquitination by CDK-regulated binding of Hct1 to the anaphase promoting complex. Science 282: 1721–1724. 9831566 10.1126/science.282.5394.1721

[11] Jaspersen SL, Charles JF, Morgan DO (1999) Inhibitory phosphorylation of the APC regulator Hct1 is controlled by the kinase Cdc28 and the phosphatase Cdc14. Curr Biol 9: 227–236. 10074450 10.1016/s0960-9822(99)80111-0

[12] Shou W, Seol JH, Shevchenko A, Baskerville C, Moazed D, et al (1999) Exit from mitosis is triggered by Tem1-dependent release of the protein phosphatase Cdc14 from nucleolar RENT complex. Cell 97: 233–244. 10219244 10.1016/s0092-8674(00)80733-3

[13] Visintin R, Hwang ES, Amon A (1999) Cfi1 prevents premature exit from mitosis by anchoring Cdc14 phosphatase in the nucleolus. Nature 398: 818–823. 10235265 10.1038/19775

[14] Traverso EE, Baskerville C, Liu Y-X, Shou W, James P, et al (2001) Characterization of the Net1 cell cycle-dependent regulator of the Cdc14 phosphatase from budding yeast. J Biol Chem 276: 21924–21931. 11274204 10.1074/jbc.M011689200

[15] Shou W, Azzam R, Chen SL, Huddleston MJ, Baskerville C, et al (2002) Cdc5 influences phosphorylation of Net1 and disassembly of the RENT complex. BMC Mol Biol 3: 3. 11960554 10.1186/1471-2199-3-3 PMC113746

[16] Yoshida S, Toh-e A (2002) Budding yeast Cdc5 phosphorylates Net1 and assists Cdc14 release from the nucleolus. Biochem Biophys Res Commun 294: 687–691. 12056824 10.1016/S0006-291X(02)00544-2

[17] Azzam R, Chen SL, Shou W, Mah AS, Alexandru G, et al (2004) Phosphorylation by cyclin B-Cdk underlies release of mitotic exit activator Cdc14 from the nucleolus. Science 305: 516–519. 15273393 10.1126/science.1099402

[18] Sullivan M, Uhlmann F (2003) A non-proteolytic function of separase links the onset of anaphase to mitotic exit. Nat Cell Biol 5: 249–254. 12598903 10.1038/ncb940 PMC2610357

[19] Queralt E, Lehane C, Novak B, Uhlmann F (2006) Downregulation of PP2A^Cdc55^ phosphatase by separase initiates mitotic exit in budding yeast. Cell 125: 719–732. 16713564 10.1016/j.cell.2006.03.038

[20] Stegmeier F, Visintin R, Amon A (2002) Separase, polo kinase, the kinetochore protein Slk19, and Spo12 function in a network that controls Cdc14 localization during early anaphase. Cell 108: 207–220. 11832211 10.1016/s0092-8674(02)00618-9

[21] Jaspersen SL, Charles JF, Tinker-Kulberg RL, Morgan DO (1998) A late mitotic regulatory network controlling cyclin destruction in *Saccharomyces cerevisiae* . Mol Biol Cell 9: 2803–2817. 9763445 10.1091/mbc.9.10.2803 PMC25555

[22] Fesquet D, Fitzpatrick PJ, Johnson AL, Kramer KM, Toyn JH, et al (1999) A Bub2p-dependent spindle checkpoint pathway regulates the Dbf2p kinase in budding yeast. EMBO J 18: 2424–2434. 10228157 10.1093/emboj/18.9.2424 PMC1171325

[23] Lee SE, Frenz LM, Wells NJ, Johnson AL, Johnston LH (2001) Order of function of the budding yeast mitotic exit-network proteins Tem1, Cdc15, Mob1, Dbf2, and Cdc5. Curr Biol 11: 784–788. 11378390 10.1016/s0960-9822(01)00228-7

[24] Mah AS, Jang J, Deshaies RJ (2001) Protein kinase Cdc15 activates the Dbf2-Mob1 kinase complex. Proc Natl Acad Sci USA 98: 7325–7330. 11404483 10.1073/pnas.141098998 PMC34667

[25] Jaspersen SL, Morgan DO (2000) Cdc14 activates Cdc15 to promote mitotic exit in budding yeast. Curr Biol 10: 615–618. 10837230 10.1016/s0960-9822(00)00491-7

[26] König C, Maekawa H, Schiebel E (2010) Mutual regulation of cyclin-dependent kinase and the mitotic exit network. J Cell Biol 188: 351–368. 20123997 10.1083/jcb.200911128 PMC2819678

[27] Sullivan M, Higuchi T, Katis VL, Uhlmann F (2004) Cdc14 phosphatase induces rDNA condensation and resolves cohesin-independent cohesion during budding yeast anaphase. Cell 117: 471–482. 15137940 10.1016/s0092-8674(04)00415-5

[28] D'Amours D, Stegmeier F, Amon A (2004) Cdc14 and condensin control the dissolution of cohesin-independent linkages at repeated DNA. Cell 117: 455–469. 15137939 10.1016/s0092-8674(04)00413-1

[29] Wang B-D, Yong-Gonzalez V, Strunnikov AV (2004) Cdc14p/FEAR pathway controls segregation of nucleolus in *S. cerevisiae* by facilitating condensin targeting to rDNA chromatin in anaphase. Cell Cycle 3: 960–967. 15190202 10.4161/cc.3.7.1003 PMC2673102

[30] D'Ambrosio C, Kelly G, Shirahige K, Uhlmann F (2008) Condensin-dependent rDNA decatenation introduces a temporal pattern to chromosome segregation. Curr Biol 18: 1084–1089. 18635352 10.1016/j.cub.2008.06.058

[31] Wang B-D, Butylin P, Strunnikov A (2006) Condensin function in mitotic nucleolar segregation is regulated by rDNA transcription. Cell Cycle 5: 2260–2267. 16969110 10.4161/cc.5.19.3292 PMC3225123

[32] Tomson BN, D'Amours D, Adamson BS, Aragon L, Amon A (2006) Ribosomal DNA transcription-dependent processes interfere with chromosome segregation. Mol Cell Biol 26: 6239–6247. 16880532 10.1128/MCB.00693-06 PMC1592809

[33] Elliott SG, McLaughlin CS (1979) Regulation of RNA synthesis in yeast III. Mol Gen Genet 169: 237–243. 372745 10.1007/BF00382269

[34] Kuilman T, Maiolica A, Scheidel N, Aebersold R, Uhlmann F (2014) Identification of Cdk targets that control cytokinesis. EMBO J. epub ahead of print: DOI10.15252/embj.201488958. 10.15252/embj.201488958 PMC4291482 25371407

[35] King MC, Lusk CP, Blobel G (2006) Karyopherin-mediated import of integral inner nuclear membrane proteins. Nature 442: 1003–1007. 16929305 10.1038/nature05075

[36] Mekhail K, Seebacher J, Gygi SP, Moazed D (2008) Role for perinuclear chromosome tethering in maintenance of genome stability. Nature 456: 667–670. 18997772 10.1038/nature07460 PMC2596277

[37] Chan JN, Poon BP, Salvi J, Olsen JB, Emili A, et al (2011) Perinuclear cohibin complexes maintain replicative life span via roles at distinct silent chromatin domains. Dev Cell 20: 867–879. 21664583 10.1016/j.devcel.2011.05.014

[38] Holt LJ, Tuch BB, Villén J, Johnson AD, Gygi SP, et al. (2009) Global analysis of Cdk1 substrate phosphorylation sites provides insights into evolution. Science 325. 10.1126/science.1172867 PMC2813701 19779198

[39] Culotti J, Hartwell LH (1971) Genetic control of the cell division cycle in yeast. III. Seven genes controlling nuclear division. Exp Cell Res 67: 389–401. 5097524 10.1016/0014-4827(71)90424-1

[40] Lyons NA, Morgan DO (2011) Cdk1-dependent destruction of Eco1 prevents cohesion establishment after S phase. Mol Cell 42: 378–389. 21549314 10.1016/j.molcel.2011.03.023 PMC3095509

[41] Defossez PA, Prusty R, Kaeberlein M, Lin SJ, Ferrigno P, et al (1999) Elimination of replication block protein Fob1 extends the life span of yeast mother cells. Mol Cell 3: 447–455. 10230397 10.1016/s1097-2765(00)80472-4

[42] Stegmeier F, Amon A (2004) Closing mitosis: The functions of the Cdc14 phosphatase and its regulation. Annu Rev Genet 38: 203–231. 15568976 10.1146/annurev.genet.38.072902.093051

[43] Shou W, Sakamoto KM, Keener J, Morimoto KW, Traverso EE, et al (2001) Net1 stimulates RNA polymerase I transcription and regulates nucleolar structure independently of controlling mitotic exit. Mol Cell 8: 45–55. 11511359 10.1016/s1097-2765(01)00291-x

[44] Rabitsch KP, Petronczki M, Javerzat J-P, Genier S, Chwalla B, et al (2003) Kinetochore recruitment of two nucleolar proteins is required for homolog segregation in meiosis I. Dev Cell 4: 535–548. 12689592 10.1016/s1534-5807(03)00086-8

[45] Chin CF, Bennett AM, Ma WK, Hall MC, Yeong FM (2012) Dependence of Chs2 ER export on dephosphorylation by cytoplasmic Cdc14 ensures that septum formation follows mitosis. Mol Biol Cell 23: 45–58. 22072794 10.1091/mbc.E11-05-0434 PMC3248903

[46] Palani S, Meitinger F, Boehm ME, Lehmann WD, Pereira G (2012) Cdc14-dependent dephosphorylation of Inn1 contributes to Inn1-Cyk3 complex formation. J Cell Sci 125: 3091–3096. 22454527 10.1242/jcs.106021

[47] Stegmeier F, Huang J, Rahal R, Zmolik J, Moazed D, et al (2004) The replication fork block protein Fob1 functions as a negative regulator of the FEAR network. Curr Biol 14: 467–480. 15043811 10.1016/j.cub.2004.03.009

[48] Queralt E, Uhlmann F (2008) Separase cooperates with Zds1 and Zds2 to activate Cdc14 phosphatase in early anaphase. J Cell Biol 182: 873–883. 18762578 10.1083/jcb.200801054 PMC2528575

[49] Tomson BN, Rahal R, Reiser V, Monje-Casas F, Mekhail K, et al (2009) Regulation of Spo12 phosphorylation and its essential role in the FEAR network. Curr Biol 19: 449–460. 19268588 10.1016/j.cub.2009.02.024 PMC2692463

[50] Waples WG, Chahwan C, Ciechonska M, Lavoie BD (2009) Putting the brake on FEAR: Tof2 promotes the biphasic release of Cdc14 phosphatase during mitotic exit. Mol Biol Cell 20: 245–255. 18923139 10.1091/mbc.E08-08-0879 PMC2613101

[51] Akiyoshi B, Biggins S (2010) Cdc14-dependent dephosphorylation of a kinetochore protein prior to anaphase in *Saccharomyces cerevisiae* . Genetics 468: 576–579. 10.1534/genetics.110.123653 PMC2998326 20923974

[52] Holt LJ, Krutchinsky AN, Morgan DO (2008) Positive feedback sharpens the anaphase switch. Nature 454: 353–357. 18552837 10.1038/nature07050 PMC2636747

[53] Huh W-K, Falvo JV, Gerke LC, Carroll AS, Howson RW, et al (2003) Global analysis of protein localization in budding yeast. Nature 425: 686–691. 14562095 10.1038/nature02026

[54] Shou W, Deshaies RJ (2002) Multiple t*elophase arrest bypassed (tab)* mutants alleviate the essential requirement for Cdc15 in exit from mitosis in *S. cerevisiae* . BMC Genet 3: 4. 11914130 10.1186/1471-2156-3-4 PMC102333

[55] Wach A, Brachat A, Pöhlmann R, Philippsen P (1994) New heterologous modules for classical or PCR-based gene disruptions in *Saccharomyces cerevisiae* . Yeast 10: 1793–1808. 7747518 10.1002/yea.320101310

[56] Knop M, Siegers K, Pereira G, Zachariae W, Winsor B, et al (1999) Epitope tagging of yeast genes using a PCR-based strategy: more tags and improved practical routines. Yeast 15: 963–972. 10407276 10.1002/(SICI)1097-0061(199907)15:10B<963::AID-YEA399>3.0.CO;2-W

[57] Wäsch R, Cross FR (2002) APC-dependent proteolysis of the mitotic cyclin Clb2 is essential for mitotic exit. Nature 418: 556–562. 12152084 10.1038/nature00856

[58] Eluère R, Offner N, Varlet I, Motteux O, Signon L, et al (2007) Compartmentalization of the functions and regulation of the mitotic cyclin Clb2 in *S. cerevisiae* . J Cell Sci 120: 702–711. 17264146 10.1242/jcs.03380

[59] Bailly E, Cabantous S, Sondaz D, Bernadac A, Simon MN (2003) Differential cellular localization among mitotic cyclins from *Saccharomyces cerevisiae*: a new role for the axial budding protein Bud3 in targeting Clb2 to the mother-bud neck. J Cell Sci 116: 4119–4130. 12972503 10.1242/jcs.00706

[60] Lindstrom DL, Gottschling DE (2009) The mother enrichment program: a genetic system for facile replicative life span analysis in *Saccharomyces cerevisiae* . Genetics 183: 413–422. 19652178 10.1534/genetics.109.106229 PMC2766306

[61] Amberg DC, Burke DJ, Strathern JN (2005) Methods in yeast genetics. Cold Spring Harbor, New York: Cold Spring Harbor Laboratory Press.

[62] Uhlmann F, Lottspeich F, Nasmyth K (1999) Sister-chromatid separation at anaphase onset is promoted by cleavage of the cohesin subunit Scc1. Nature 400: 37–42. 10403247 10.1038/21831

[63] Foiani M, Marini F, Gamba D, Lucchini G, Plevani P (1994) The B subunit of the DNA polymerase α-primase complex in *Saccharomyces cerevisiae* executes an essential function at the initial stage of DNA replication. Mol Cell Biol 14: 923–933. 8289832 10.1128/mcb.14.2.923-933.1994 PMC358447

