# Supplementary figures and images for: Nur1 Dephosphorylation Confers Positive Feedback to Mitotic Exit Phosphatase Activation in Budding Yeast
Source: PLoS Genet. 2015 Jan 8;11(1):e1004907. doi: 10.1371/journal.pgen.1004907 (PMC4287440; doi:10.1371/journal.pgen.1004907)

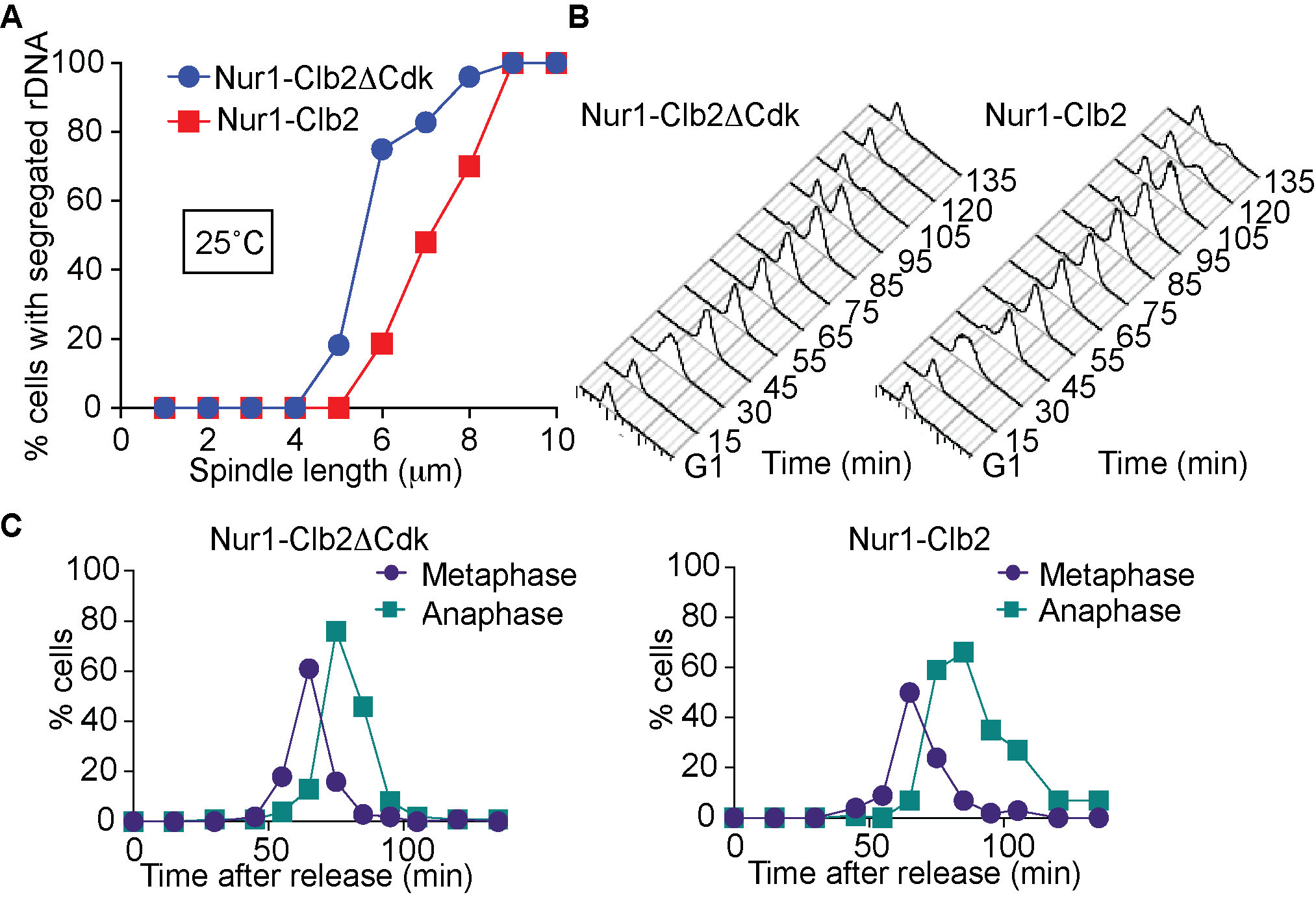

Supplement: S1 Fig — An rDNA segregation and mitotic exit delay in Nur1-Clb2 cells at a permissive temperature of 25°C. A. rDNA segregation is delayed in Nur1-Clb2, as compared to Nur1-Clb2ΔCdk cells. Completion of rDNA segregation is plotted as a function of spindle length. B. Progression through mitosis is delayed in Nur1-Clb2 cells, as seen by FACS analysis of DNA content of cultures passing through a synchronous cell cycle following α-factor arrest and release at 25°C. C. Cells from the timecourse shown in B were processed to visualize spindle microtubules by indirect immunofluorescence using an antibody against α-tubulin. A mitotic exit delay in Nur1-Clb2 cells is manifest by the longer persistence of cells containing elongated anaphase spindles. (TIF) [file pgen.1004907.s001.tif]

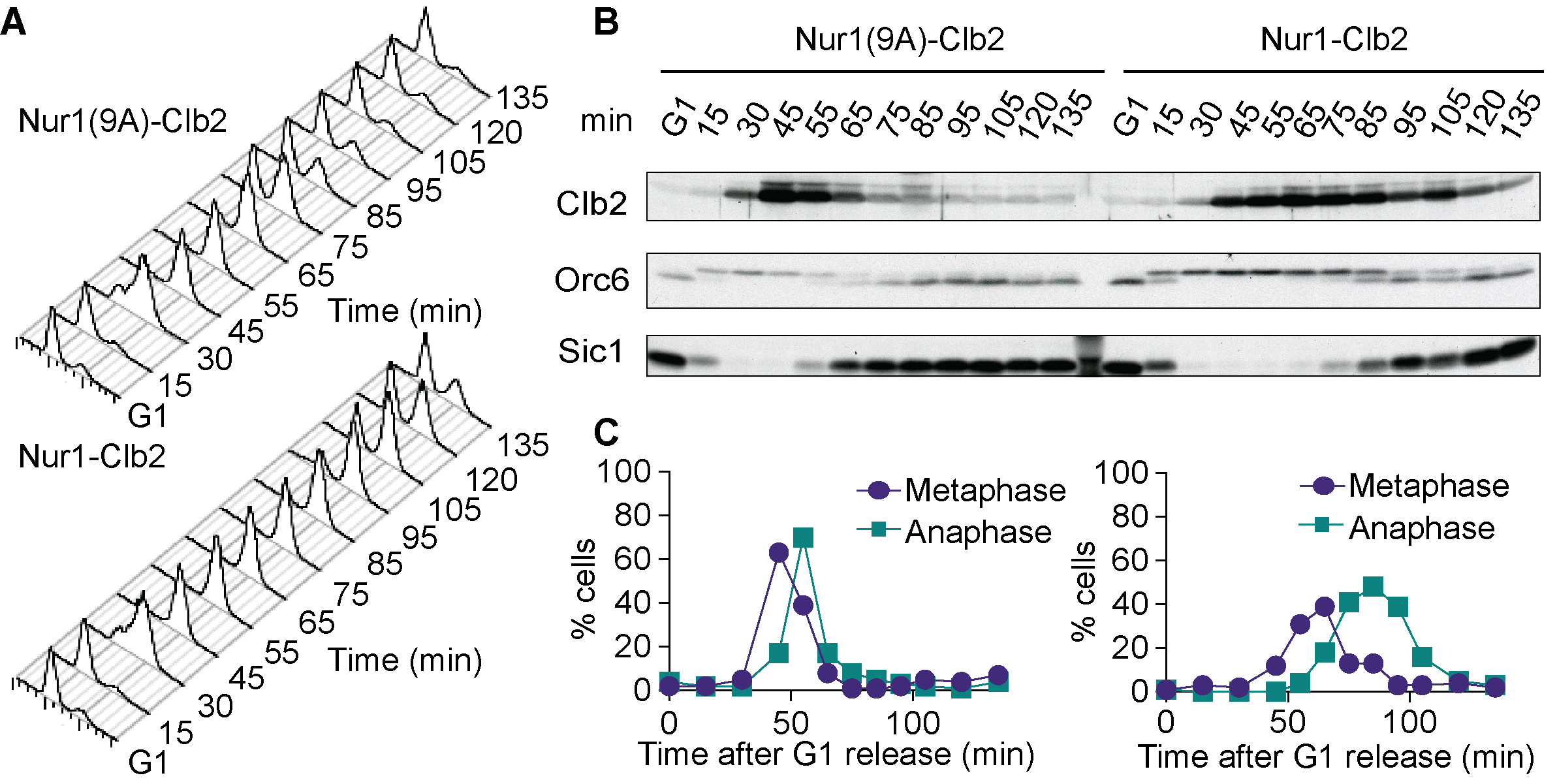

Supplement: S2 Fig — Nur1 phosphorylation is responsible for the mitotic exit delay seen in Nur1-Clb2 cells. Nur1-Clb2 and Nur1(9A)-Clb2 cells were synchronized in G1 by α-factor treatment and released to progress through the cell cycle at 36°C, before being rearrested in the following G1. At time points throughout the cell cycle we monitored, A. cell cycle progression by FACS analysis of DNA content. B. levels of the cell cycle markers Clb2, Sic1 and Orc6 by Western blot analysis, and C. the percentages of cells displaying metaphase (1–3 µm) or anaphase (>3 µm) spindles. 100 cells were counted at each time point. (TIF) [file pgen.1004907.s002.tif]

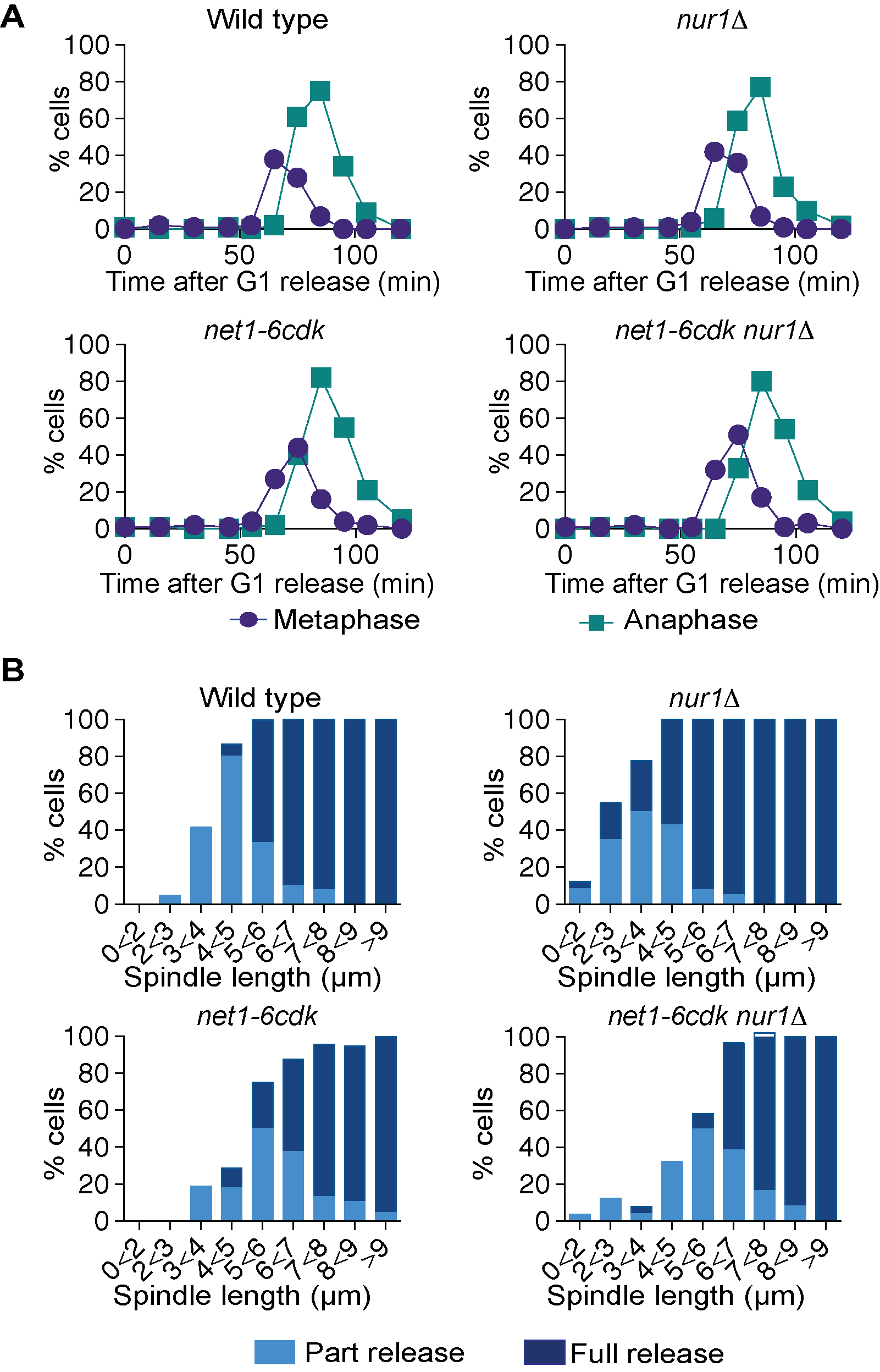

Supplement: S3 Fig — Nur1's effect on Cdc14 is dependent on Net1's phosphorylation status. A. Progression through mitosis of wild type, nur1Δ, net1-6cdk and nur1Δ net1-6cdk cells was measured by counting percentages of cells displaying metaphase (1–3 µm) or anaphase (>3 µm) spindles during synchronous cell cycle progression following α-factor arrest and release. B. Quantification of Cdc14 release versus spindle length, as in Figure 5, during the experiment above. (TIF) [file pgen.1004907.s003.tif]
