# Supplementary material for: Nur1 Dephosphorylation Confers Positive Feedback to Mitotic Exit Phosphatase Activation in Budding Yeast
Source: PLoS Genet. 2015 Jan 8;11(1):e1004907. doi: 10.1371/journal.pgen.1004907 (PMC4287440; doi:10.1371/journal.pgen.1004907)
Supplement: S1 Table — List of yeast strains used in this study. (DOCX) [file pgen.1004907.s004.docx]

| Name | Genotype | *MAT* |
| --- | --- | --- |
| Y141  (w303) | *ade2-1, trp1-1, can1-100, leu2-3,112, his3-11,15, ura3-52, GAL, psi^+^* | a |
| Y4652 | *NUR1-PK_3_::TRP1* | a |
| Y4653 | *cdc14-1, NUR1-PK_3_::TRP1* | a |
| Y4565 | *trp1::TRP1-CRE-EBD78* | a |
| Y4654 | *nur1-clb2::URA3, trp1::TRP1-CRE-EBD78* | a |
| Y4655 | *nur1-clb2Δcdk::URA3, trp1::TRP1-CRE-EBD78* | a |
| Y4656 | *RDN1::ADE2, rad5* | a |
| Y4657 | *RDN1::ADE2, rad5, trp1::TRP1-CRE-EBD78* | a |
| Y4658 | *RDN1::ADE2, rad5, nur1-clb2::URA3, trp1::TRP1-CRE-EBD78* | a |
| Y4659 | *RDN1::ADE2, rad5, nur1-clb2Δcdk::URA3, trp1::TRP1-CRE-EBD78* | a |
| Y4660 | *NET1-YFP::HIS3,* *nur1-clb2::URA3, trp1::TRP1-CRE-EBD78* | a |
| Y4661 | *NET1-YFP::HIS3,* *nur1-clb2Δcdk::URA3, trp1::TRP1-CRE-EBD78* | a |
| Y4662 | *CDC14-GFP::HIS3,* *nur1-clb2::URA3, trp1::TRP1-CRE-EBD78* | a |
| Y4663 | *CDC14-GFP::HIS3,* *nur1-clb2Δcdk::URA3, trp1::TRP1-CRE-EBD78* | a |
| Y4664 | *nur1Δ::LEU2* | a |
| Y4665 | *nur1(9A)::LEU2* | a |
| Y4666 | *nur1Δ::LEU2, CDC14-GFP::TRP1* | a |
| Y4667 | *nur1(9A)::LEU2, CDC14-GFP::TRP1* | a |
| Y4668 | *nur1Δ::LEU2, CDC14-GFP::TRP1, spo12Δ::HIS3* | a |
| Y4669 | *nur1(9A)::LEU2, CDC14-GFP::TRP1, spo12Δ::HIS3* | a |
| Y4670 | *CDC14-GFP::TRP1, spo12Δ::HIS3* | a |
| Y4671 | *CDC14-GFP::TRP1* | a |
| Y4672 | *nur1(9A)-clb2::URA3, trp1::TRP1-CRE-EBD78* | a |
| Y4673 | *nur1(9A)-clb2Δcdk::URA3, trp1::TRP1-CRE-EBD78* | a |
| Y4675 | *nur1-clb2::URA3, trp1::TRP1-CRE-EBD78, CDC14^TAB6-1^-Pk_3_::LEU2* | a |
| Y4676 | *nur1-clb2Δcdk::URA3, trp1::TRP1-CRE-EBD78, CDC14^TAB6-1^-Pk_3_::LEU2* | a |
| Y844 | *dbf2-2* | α |
| Y145 | *cdc15-2* | a |
| Y4677 | *dbf2-2, nur1Δ::LEU2* | α |
| Y4678 | *cdc15-2, nur1Δ::LEU2* | a |
| Y4679 | *NET1-myc_9_::TRP1, CDC14-HA_6_::HIS3, NUR1-Pk_3_::LEU2* | a |
| Y2785 | *net1Δ::S.p.HIS5, net1-6Cdk-TEV-myc9::TRP1, CDC14-Pk_9_-Kan^R^* | a |
| Y2786 | *CDC14-Pk_9_-Kan^R^* | a |
| Y4705 | *net1Δ::S.p.HIS5, net1-6Cdk-TEV-myc9::TRP1, CDC14-Pk_9_-Kan^R^, nur1Δ::LEU2* | a |
| Y4706 | *CDC14-Pk_9_-Kan^R^, nur1Δ::LEU2* | a |

Table S1: List of *Saccharomyces cerevisiae* strains used in this study
